# A Hierarchical Framework and Marginal Return Optimization for Dynamic Task Allocation in Heterogeneous UAV Networks

**DOI:** 10.3390/s25216676

**Published:** 2025-11-01

**Authors:** Anxin Guo, Zhenxing Zhang, Ao Wu, Qi Li, Leyan Li, Rennong Yang

**Affiliations:** 1 Air Traffic Control and Navigation School, Air Force Engineering University, Xi’an 710000, China; anxing0519@163.com (A.G.);; 2 Science and Technology on Complex Aviation Systems Simulation Laboratory, Beijing 100000, China

**Keywords:** hierarchical framework, mission chain, heterogeneous UAVs, dynamic task allocation, sensor-effector coordination, heuristic optimization

## Abstract

The coordination of heterogeneous Unmanned Aerial Vehicles (UAVs) for complex, multi-stage tasks presents a significant challenge in robotics and autonomous systems. Traditional linear models often fail to capture the emergent synergistic effects and dynamic nature of multi-agent collaboration. To address these limitations, this paper proposes a novel hierarchical framework based on a Mission Chain (MC) concept. We systematically define and model key elements of multi-agent collaboration, including Mission Chains (MCs), Execution Paths (EPs), Task Networks (TNs), and Solution Spaces (SSs), creating an integrated theoretical structure. Based on this framework, we formulate the problem as a Sensor–Effector–Target Assignment challenge and propose a Marginal Return-Based Heuristic Algorithm (MRBHA) for efficient dynamic task allocation. Simulations demonstrate that our proposed MRBHA achieves a substantially higher total expected mission value—outperforming standard greedy and random assignment strategies by 14% and 77%, respectively. This validates the framework’s ability to effectively capitalize on synergistic opportunities within the UAV network. The proposed system provides a robust and scalable solution for managing complex missions in dynamic environments, with potential applications in search-and-rescue, environmental monitoring, and intelligent logistics.

## 1. Introduction

The proliferation of Unmanned Aerial Vehicles (UAVs) in multi-agent systems has unlocked new possibilities across a wide range of applications, including environmental monitoring, disaster response, precision agriculture, and intelligent logistics [1,2,3,4,5]. The use of heterogeneous UAVs—teams composed of agents with diverse capabilities (e.g., some equipped for sensing, others for carrying out specific actions)—offers enhanced flexibility, robustness, and efficiency compared to homogeneous swarms [6,7,8,9]. However, effectively coordinating these heterogeneous assets to perform complex, multi-stage missions in dynamic environments remains a significant challenge. The core of this challenge lies in solving the dynamic task allocation problem: assigning the right agent to the right task at the right time to maximize overall mission success [10,11,12,13,14]. To manage the complexity of these missions, researchers often model them as a sequence of dependent sub-tasks, frequently termed a “Task Execution Chain” [15,16].

Current research on UAV task allocation often falls into two primary categories, each with inherent limitations in addressing complex, multi-stage missions.

The first category focuses on solving single-stage assignment problems, treating sensing and effecting as isolated sub-problems. For instance, significant work has been performed on optimizing sensor coverage [17] or assigning effector UAVs to specific targets [18,19,20]. While these methods are effective for their designated tasks, they inherently fail to optimize the end-to-end mission workflow. They overlook the critical dependencies between stages—for example, how an initial, high-quality sensor assignment can dramatically increase the probability of success for a subsequent effector action. This compartmentalized approach prevents the system from achieving true synergistic collaboration.

The second category employs general-purpose optimization algorithms—such as genetic algorithms [12,21,22], particle swarm optimization, or reinforcement learning [23,24,25,26,27]—to solve the allocation problem holistically. Although powerful, these approaches often treat the problem as a “black box.” They lack a tailored conceptual structure that explicitly models the multi-stage dependencies and synergistic effects between heterogeneous agents. Consequently, the potential for emergent collaboration is handled implicitly rather than being a core component of the problem formulation, often leading to computationally expensive searches and sub-optimal resource allocation.

This review of the state of the art reveals a critical gap: the absence of a unified, hierarchical framework that can systematically model the entire mission workflow and translate high-level strategic goals into an optimized, low-level operational plan. This paper bridges this gap by proposing a novel hierarchical framework. We refine and formalize the general concept of a “Task Execution Chain” into our structured Mission Chain (MC), which serves as the strategic backbone for modeling and optimizing. Our framework decomposes a mission into a three-level hierarchy: the Mission Chain (MC), which defines the overall strategic workflow; the Execution Path (EP), representing a specific combination of agents capable of completing the mission for a single target; and the Execution Task (ET), which represents the fundamental action performed by an individual agent, such as ‘sensor S1 tracks target T2’ or ‘effector E3 services target T2’. This structure provides a clear and scalable way to manage mission complexity.

The primary contributions of this work are threefold:

1. The introduction of a formal hierarchical framework (MC-EP-ET) that systematically models complex, multi-stage tasks, providing a structured and scalable foundation for coordinating heterogeneous UAV teams.

2. The formulation of a Sensor–Effector–Target Assignment (S-ETA) model, a novel mathematical programming approach that captures the unique challenge of assigning specialized sensor and effector agents to maximize total expected mission value [28,29].

3. The development of an efficient Marginal Return-Based Heuristic Algorithm (MRBHA), specifically designed to solve the S-ETA problem by quantitatively evaluating and capitalizing on the synergistic contributions of each potential sensor–effector–target triad.

Collectively, these contributions provide an end-to-end solution that bridges the gap between high-level mission strategy and low-level agent execution, enabling a more intelligent and synergistic allocation of resources than previously achievable with disconnected or black-box methods.

The remainder of this paper is organized as follows. Section 2 introduces the complete hierarchical framework, from the Mission Chain to the Solution Space. Section 3 conceptualizes the operational scenario and defines the abstract problem. Section 4 details the S-ETA mathematical programming model and its constraints. Section 5 presents the proposed MRBHA in detail. Section 6 describes the experimental setup, presents the assignment and comparative results, and provides an in-depth analysis of the algorithm’s performance. Finally, Section 7 provides concluding remarks and discusses future research directions.

## 2. The Hierarchical Mission Chain Framework

This section introduces a conceptual framework for modeling complex, multi-stage autonomous missions. Inspired by the need for structured coordination in multi-agent systems, our work systematically defines and standardizes a hierarchical “Mission Chain” concept. We analyze the core components and logical connections at each level of this hierarchy using structured methods. The result is a unified theoretical framework for addressing dynamic targets in complex operational environments. This framework provides essential theoretical support for enhancing the synergistic performance of multi-agent systems and improving the robustness of automated decision-making.

### 2.1. The Mission Chain

**Definition 1: Mission Chain (MC).** A Mission Chain is a logical sequence of phases required to successfully service a single target. Mathematically, it is defined as an ordered set of mission tasks (MT):

(1) $$MC=[{\mathrm{MT}}_{1},{\mathrm{MT}}_{2},\cdots ,MT_{n}]$$

In this equation, MC represents the Mission Chain, $MT_{i}$ denotes the ith operational phase, and n is the total number of sequential phases required to complete the mission for that target.

**Figure 1:**
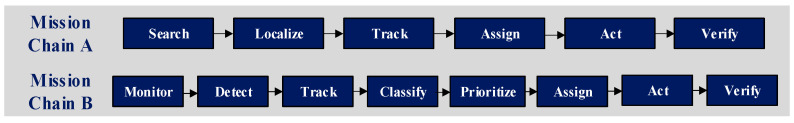
Example phase structures for different types of Mission Chains.

As demonstrated in Figure 1, the structure of a Mission Chain is adaptable to specific application requirements. For example, a time-critical search-and-rescue mission might employ a concise six-phase sequence: (1) area search, (2) point of interest localization, (3) object classification, (4) task prioritization, (5) action execution (e.g., payload delivery), and (6) outcome verification. In contrast, a persistent environmental monitoring mission might utilize a more extensive eight-phase protocol, including continuous surveillance, anomaly detection, data collection, and long-term tracking. This tailored approach allows the framework to accommodate diverse mission objectives and environmental constraints through modular phase adjustments.

The probability $P(S_{MC})$ of a successful mission for a given *MC* is the product of the success probabilities of its constituent phases:

(2) $$P(S_{MC})=\prod_{i=1}^{n}P(S_{MT_{i}})$$

In this equation, $P(S_{MT_{i}})$ represents the probability of success for phase $MT_{i}$. The *MC* structure is inherently sequential, requiring each phase to be completed successfully before the next can be initiated.

It is assumed here that the success of each mission phase is a probabilistically independent event. This is a common simplification for initial framework development to ensure model tractability. Future extensions could incorporate conditional probabilities to model dependencies between phases.

### 2.2. The Execution Path

**Definition 2: Execution Path (EP).** An Execution Path is a specific instantiation of a Mission Chain, where each phase of the chain is assigned to a concrete set of agents. While the Mission Chain defines what needs to be done, the Execution Path defines who does it and how. It maps the abstract sequence of tasks to specific system resources (agents, sensors, effectors) over time. Its mathematical definition is a sequence of pairs:

(3) $$EP=\left\{\left.(MT_{1},A_{1}),(MT_{2},A_{2}),\cdots ,(MT_{n},A_{n})|T\right\}\right.$$

where $\;(MT_{i},\;A_{i})$ is a pair representing that mission phase i  is executed by the agent set $A_{i}$. $A_{i}$ is the set of one or more agents (e.g., UAVs, ground stations) assigned to phase $MT_{i}$. Agents can be platforms, personnel, or equipment. T represents the single, specific target being addressed by the entire Execution Path.

To make the distinction intuitive, one can use a simple analogy: if the Mission Chain (MC) is a generic recipe for baking a cake (e.g., “mix ingredients, bake, frost”), then an Execution Path (EP) is that specific recipe being carried out by a particular person using specific tools (e.g., “Chef John uses a KitchenAid mixer, bakes in a Bosch oven, and frosts with a Wilton spatula”). The MC is the ‘what,’ and the EP is the ‘who’ and ‘how.’

**Figure 2:**
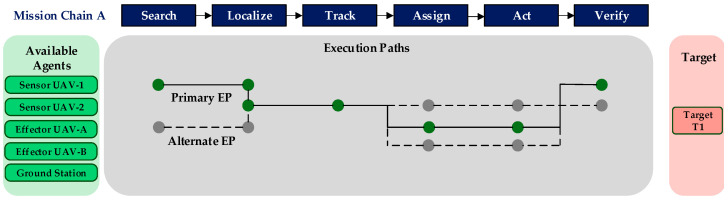
Schematic diagram of primary and alternative EPs for a Mission Chain.

A schematic diagram illustrating the Execution Path concept is presented in Figure 2. The diagram shows how a single Mission Chain (top bar) can be realized by different combinations of available agents (left box). The green path represents a potential primary EP, utilizing a preferred set of agents. The gray paths represent alternative EPs, which provide system redundancy and adaptability by assigning tasks to other available agents. Each node on a path signifies a specific agent performing a specific phase of the mission, visually mapping the collaborative workflow from start to finish.

The probability of successful execution for a given EP, denoted as $P(S_{EP})$ is the product of the conditional success probabilities for each phase:

(4) $$P(S_{EP})=\prod_{i=1}^{n}P(S_{MT_{i}}\;|\;A_{i},T)$$

where $P(S_{MT_{i}}|A)$ is the probability of successfully completing phase $MT_{i}$ with the assigned agent set $A_{i}$ for target T. The analysis of an EP’s viability involves several key factors:

(1) Probabilistic Success: The overall success of an EP is inherently probabilistic, depending on the reliability and performance of the assigned agents in each phase.

(2) Resource Allocation: Each EP represents a unique allocation of the system’s finite resources. The optimization goal is often to find the EP with the highest probability of success for a given resource cost.

(3) Temporal Constraints: An EP is only viable if the cumulative time to execute all its phases meets the mission’s temporal requirements, especially for time-sensitive targets.

(4) Coordination and Communication: A valid EP requires robust communication links between agents to facilitate information flow, such as passing target coordinates from a sensor agent to an effector agent.

### 2.3. The Task Network

**Definition 3: Task Network (TN).** A Task Network represents the complete set of all viable Execution Paths for servicing a single target. It encapsulates the system’s total collaborative capacity and all possible ways to address one specific target, given the available resources. The mathematical definition is:

(5) $$TN_{T}=\left\{\left.EP_{1},EP_{2},\cdots ,EP_{m}\right\}\right.$$

where $TN_{T}$ is the Task Network for a specific target T and m is the total number of unique, valid EPs available for that target.

**Figure 3:**
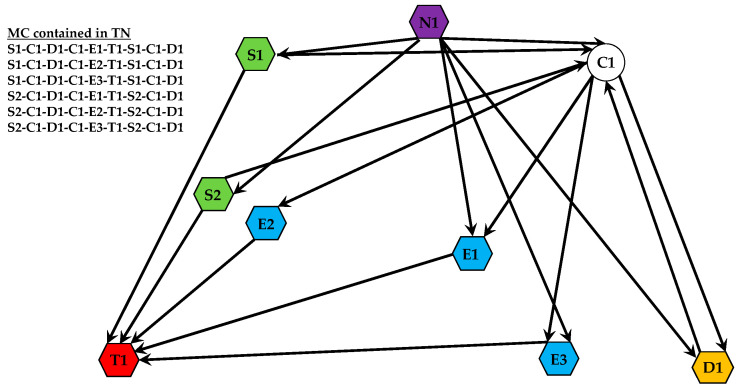
A conceptual diagram of a TN for a single target.

A TN can be conceptualized as a network composed of all feasible EPs for a given target. This construct allows for a systematic analysis of different collaborative strategies, enabling the selection of the most efficient path based on criteria such as success probability or resource cost. As illustrated in Figure 3, the Task Network for a target is formed by the connections between all relevant system assets. In this example, S1 and S2 are sensor platforms, E1, E2, and E3 are effector platforms, C1 is a communication node, and D1 is a decision-making module. The entire web of connections represents the TN, while a single path through it (e.g., from a sensor to an effector to the target T1) constitutes one EP.

### 2.4. The Mission Plan

**Definition 4: Mission Plan (MP).** A Mission Plan is a specific, conflict-free set of Execution Paths selected to service multiple targets simultaneously. According to this definition, its mathematical notation can be expressed as:

(6) $$MP=\left\{\left.EP_{1}|T_{1},EP_{2}|T_{2},\cdots ,EP_{s}|T_{s}\right\}\right.$$

where $EP_{i}\;|\;T_{i}$ represents the specific Execution Path chosen to service target T_i_ and s is the total number of targets being serviced in the plan.

**Figure 4:**
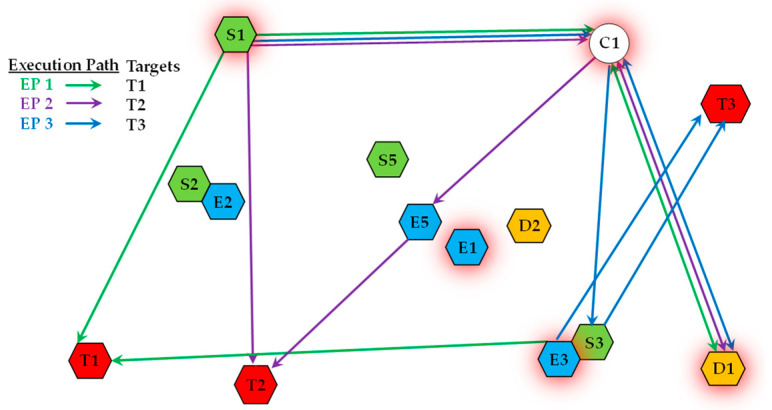
A schematic of an MP coordinating multiple EPs.

An *MP* represents a comprehensive course of action for the entire system. It enables agents to reuse resources across different Execution Paths, thereby leveraging synergistic benefits. As shown in Figure 4, an MP composed of three distinct Execution Paths is presented. These paths are used to handle targets T1, T2, and T3, respectively. The figure illustrates how multiple platforms can be involved in different EPs. For instance, the communication platform C1 and decision-making platform D1 support all three paths, while sensor S1 contributes to the EPs for both T1 and T2, demonstrating efficient resource sharing.

The process of constructing a valid MP involves the following key steps:

(1) Resolving Resource Conflicts: Ensure that no agent is assigned to more than one task simultaneously, or that any resource sharing is explicitly managed.

(2) Determining Temporal Relationships: When resources are reused across different EPs, establish the sequential order in which tasks should be performed to respect operational constraints.

### 2.5. The Solution Space

**Definition 5: Solution Space (SS).** The Solution Space is the collection of all feasible Mission Plans in a given operational environment. It represents the entirety of the system’s potential strategies for addressing all identified targets. The Solution Space can be formally expressed as:

(7) $$SS=\left\{\left.MP_{1},MP_{2},\cdots ,MP_{s}\right\}\right.$$

where SS represents the Solution Space, and $MP_{i}$ denotes the ith feasible Mission Plan. In some cases, if the missions are independent and do not share resources, the overall Solution Space can be viewed as the union of several independent sub-spaces:

(8) $$SS=SS_{1}\cup SS_{2}\cup \cdots SS_{k}$$

**Figure 5:**
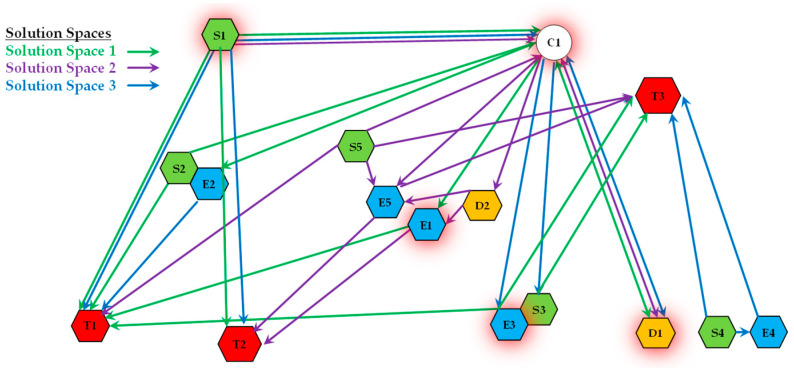
Schematic diagram of a Solution Space.

Figure 5 provides a visual representation of a Solution Space. In this diagram, nodes represent operational resources (e.g., sensors, effector agents) and targets. The paths in different colors (e.g., red, green, blue) each represent a distinct Mission Plan for accomplishing a set of tasks. Critically, we can observe that some resource nodes are shared by multiple paths (i.e., multiple MPs). This visualization clearly illustrates the core concept of a Solution Space: a complex web of overlapping and interconnected mission plans that draw from a common pool of resources.

### 2.6. System-of-Systems Operational Webs

**Definition 6: System-of-Systems Operational Webs (SoSOWs)**. A System-of-Systems Operational Web is the collection of all Solution Spaces from multiple, potentially independent, operational systems that are coordinated at a higher level. An SoSOW facilitates the optimal allocation of resources across different systems to achieve broader, collaborative objectives. It enables the integration of diverse systems, assets, and resources within a large-scale operational environment. The mathematical definition is expressed as:

(9) $$SoSOW\;=\;\{SS_{1},SS_{2},\;\cdots ,\;SS_{k}\}$$

where $SS_{i}$ denotes the Solution Space for operational subsystem *i*.

Building a System-of-Systems Operational Web involves the following key principles:

(1) Integration of Multiple Solution Spaces: Integrating dispersed Solution Spaces from heterogeneous systems to form a unified and interconnected operational network.

(2) Cross-Domain Synergy: Ensuring that resources and capabilities from multiple operational domains (e.g., aerial, ground, maritime) can be synergized to achieve complex, multi-domain objectives.

(3) High-Level Resource Optimization: Optimizing the allocation of high-value or shared resources across different systems to support the efficient operation of the entire System-of-Systems.

**Figure 6:**
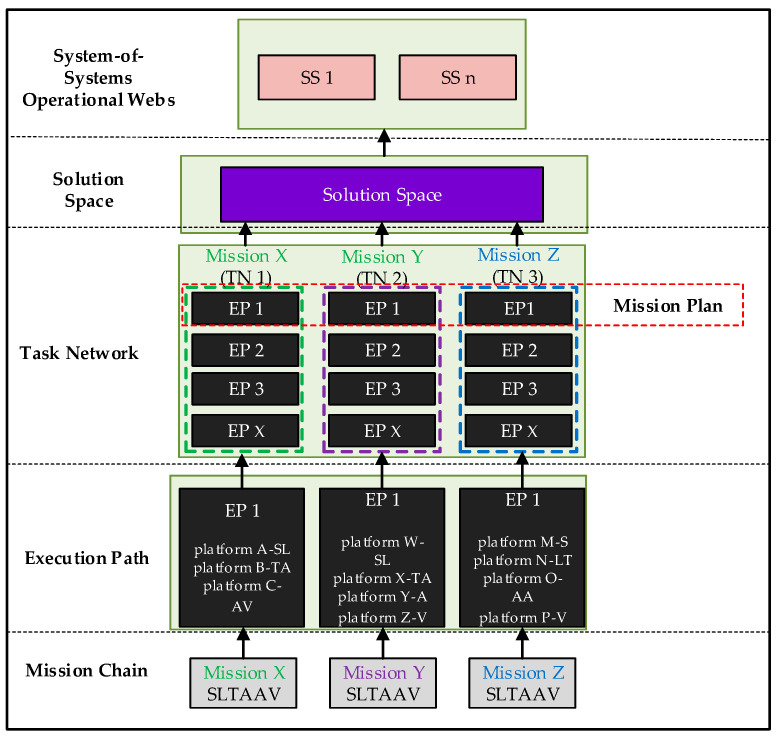
The complete hierarchical framework.

In summary, the complete hierarchical framework developed in this paper is shown in Figure 6.

## 3. Operational Scenario Conceptualization

### 3.1. Scenario Description

**Figure 7:**
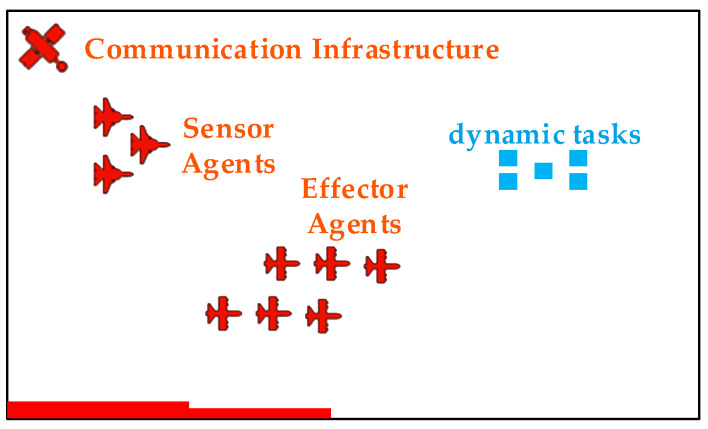
Schematic diagram of operational environment scenario.

To validate our proposed framework, we define a dynamic multi-agent tasking scenario, as conceptually illustrated in Figure 7. In this scenario, a heterogeneous fleet of Unmanned Aerial Vehicles (UAVs) is deployed to monitor a designated operational area. The objective is to detect, identify, and respond to a series of time-sensitive events or tasks that appear dynamically within this area.

The UAV fleet consists of two specialized types of agents:

(1) Sensor Agents (S): Reconnaissance UAVs (e.g., RUAS) equipped with advanced sensors to perform search, localization, and tracking of emergent tasks.

(2) Effector Agents (E): UAVs equipped to perform a specific action to “service” a task. This action could be delivering an emergency payload, collecting a specific data sample, or neutralizing a localized hazard.

The operational environment also contains critical infrastructure, including the following:

(1) Decision-Making Nodes (D): Ground stations or command modules responsible for processing sensor data and running optimization algorithms to generate Mission Plans.

(2) Communication Infrastructure (C): A network of relays and satellites ensuring robust data links between all agents and nodes.

(3) Tasks/Targets (T): Time-sensitive events that require servicing, each with an associated priority or value.

This scenario represents a classic dynamic Sensor–Effector–Target Assignment (SETA) problem, where the core challenge is to optimally allocate sensor and effector resources to maximize the total value of completed tasks in real-time.

It is important to clarify the platform context for this scenario. While the hierarchical framework itself is platform-agnostic, the S-ETA mathematical model and the assumptions underlying our simulation (e.g., agents being readily assignable to tasks without complex trajectory constraints) are most directly applicable to multirotor UAV platforms. These platforms, such as quadcopters or hexacopters, possess the ability to hover, perform vertical take-offs and landings (VTOL), and exhibit high maneuverability within a constrained operational area. This makes them well-suited for the dynamic “stop-and-stare” sensing and precise effector tasks modeled in our problem, whereas fixed-wing platforms would introduce additional complexities like minimum airspeed and turning radii that are considered outside the scope of this particular study.

### 3.2. Network Representation of the Scenario

**Figure 8:**
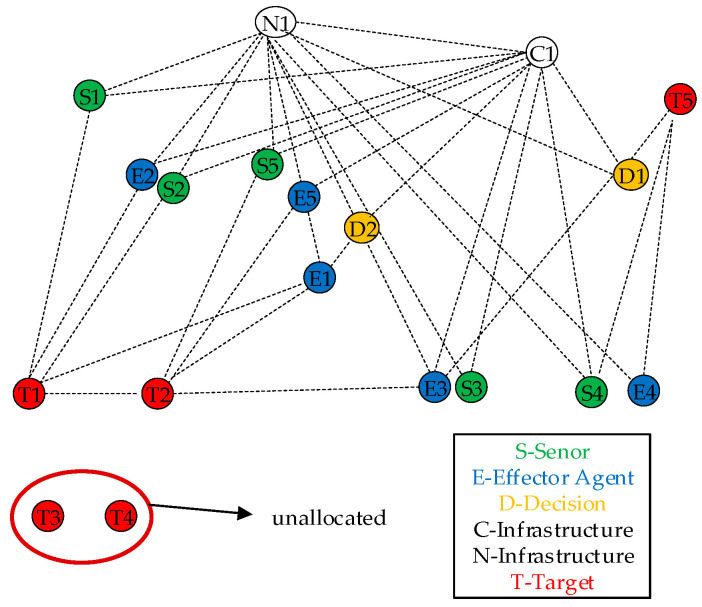
Network representation of the operational scenario.

The operational scenario can be modeled as a dynamic graph, as shown in Figure 8. Each entity—sensor, effector, decision node, communication link, and target—is represented as a node in the network. The edges between nodes represent potential interactions, such as information flow, command-and-control relationships, or resource dependencies.

For example, a sensor node S can be connected to a target node T to represent a detection or tracking action. An effector node E connects to a target node T to represent a task servicing action. Both S and E nodes are connected to decision D and communication C nodes, which are essential for coordinated operations. The goal of the system is to form effective Execution Paths (e.g., S→D→E→T) that constitute a globally optimal Mission Plan within the Solution Space.

### 3.3. Abstract Problem Formulation

Assuming the decision-making (D) and communication (C) infrastructure provides real-time support, the core problem simplifies to forming Mission Chains by optimally matching sensor agents to targets and effector agents to the same targets.

**Figure 9:**
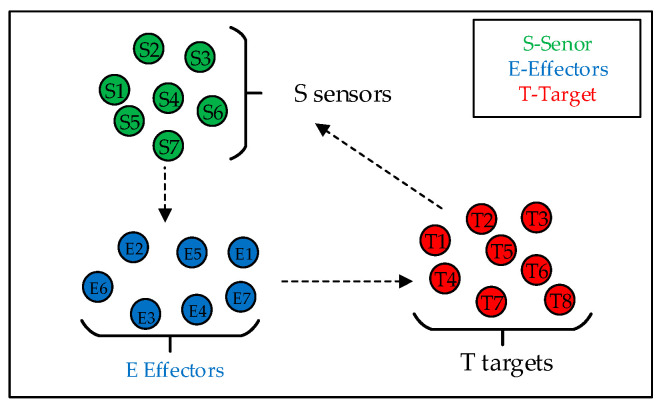
Abstract problem conceptualization.

Therefore, the operational concept is abstracted in Figure 9. A fleet of S sensor agents is tasked with discovering and tracking T dynamic targets. Guided by the information provided by the sensors, the system must then assign E effector agents to service these targets. The agents are distributed throughout the operational area. The fundamental challenge is to solve this Sensor–Effector–Target Assignment problem to maximize mission effectiveness. For simplicity in our model, we will henceforth refer to the reconnaissance platforms as sensors and the task-servicing platforms as effectors.

## 4. The S-ETA Mathematical Programming Model

Given the time-sensitive nature of the tasks and the narrow window of opportunity for execution, a centralized assignment approach is adopted. Each incoming target has an associated value, and multiple sensors and effector agents can be assigned to a single target. However, each sensor can only track a single target at a time, and each effector agent can only service a single target at a time.

Denote the sensor–target assignment scheme (STA scheme) as $Y={\left[y_{ik}\right]}_{S\times T}$, where $y_{ik}$ is the STA variable (i=1,2,⋯,S, k=1,2,⋯,T) for the ith sensor and the kth target, and $y_{ik}=1$ if the ith sensor is assigned to the kth target, and $y_{ik}=0$ otherwise.

Denote $Z={\left[\;z_{jk}\right]}_{E\times T}$ to denote the (Effector Agent -target assignment scheme) ETA scheme, where $z_{jk}$ is the ETA variable (j=1,2,⋯,E) with respect to the jth Effector Agent and the kth target, and $z_{jk}=1$ if the jth Effector Agent is assigned to the kth target, otherwise $z_{jk}=0$. For clarity, a comprehensive list of all symbols and their descriptions is provided in Appendix A (Table A1).

Based on the definitions above, the S-ETA optimization problem is formally stated as follows:

Maximize:

(10) $$J(Y,\;Z)=\sum_{k=1}^{T}v_{k}\cdot P_{serv}(k)=\sum_{k=1}^{T}v_{k}\cdot [1-\;{\prod_{i=1}^{s}\left(1-p_{ik}\right)}^{y_{ik}}]\cdot [1-\;{\prod_{j=1}^{E}\left(1-q_{jk}\right)}^{z_{\mathrm{jk}}}]$$

Subject to:

$$\begin{array}{ccccc} C1 & \sum_{k=1}^{T}y_{ik}\leq 1 & \forall i\in \left\{1,2,\cdots ,S\right\} & & \\ C2 & \sum_{k=1}^{T}z_{ik}\leq 1 & \forall j\in \left\{1,2,\cdots ,E\right\} & & \\ C3 & \sum_{k=1}^{S}y_{ik}\leq m_{k} & \forall k\in \left\{1,2,\cdots ,T\right\} & & \\ C4 & \sum_{k=1}^{E}z_{jk}\leq n_{k} & \forall k\in \left\{1,2,\cdots ,T\right\} & & \\ C5 & y_{ik},z_{ik}\in \{0,1\} & & & \end{array}$$

The objective function, derived from Equation (10), seeks to maximize the sum of expected values across all targets. The constraints are defined as follows: Constraint (C1) and (C2) ensure that each sensor and effector agent are assigned to at most one target. Constraints (C3) and (C4), limit the maximum number of agents that can be cooperatively assigned to a single target based on its priority (v_k_). Finally, (C5) defines the decision variables as binary.

The formulation of our objective function relies on an OR fusion rule for calculating group success probability (i.e., one minus the product of individual failure probabilities). This choice is fundamentally motivated by the operational nature of the tasks in our scenario. A mission phase, such as tracking or servicing a target, is typically deemed successful if at least one of the assigned agents succeeds. For instance, a target is considered successfully tracked if just one sensor maintains a stable track lock, and it is considered serviced if at least one effector successfully completes its action.

An alternative AND fusion rule, which would require all assigned agents to succeed simultaneously ($P_{group}=\;\Pi \;p_{individual}$), would be overly restrictive and unrealistic for this application. It would fail to model the redundancy and collaborative enhancement that multi-agent assignment is intended to provide. Similarly, a majority rule would not be appropriate, as it would undervalue the critical contribution of a single, highly capable agent whose individual success should be sufficient to complete the task. Therefore, the OR fusion rule most accurately reflects the desired collaborative outcome in our S-ETA problem.

## 5. The Proposed MRBHA

### 5.1. Supporting Decision Matrix

In a dynamic multi-agent system, sensor and effector agents can be flexibly combined to form Execution Paths. A key challenge is representing the assignment of a sensor–effector–target triad. To facilitate this, we introduce a three-dimensional ternary matrix $X={\left[x_{ijk}\right]}_{S\times E\times T}$ as an auxiliary decision matrix.

Here, $x_{ijk}=1$ if the specific triad (sensor i, effector j, target k) is selected for an Execution Path, and $x_{ijk}=0$ otherwise. This matrix provides a granular view of the complete assignment plan. The original binary decision matrices, Y (sensor–target) and Z (effector–target), can be directly derived from X, as shown in Equation (11).

(11) $$\left\{\begin{array}{c} y_{ik}=\underset{\;j}{\max}\;\{x_{ijk}\}\\ z_{jk}=\underset{\;i}{\max}\;\{x_{ijk}\} \end{array}\right.$$

Equation (11) illustrates how the binary assignment matrices are derived from X. Specifically, $y_{ik}$ is set to 1 if sensor *i* is assigned to target *k* as part of any triad (i.e., for any effector *j*). Similarly, $z_{jk}$ is set to 1 if effector agent *j* is assigned to target *k* (with any sensor *i*).

### 5.2. Constraint Processing

To ensure that the generated solutions adhere to the resource constraints, we introduce several auxiliary variables to track resource utilization. To manage constraints (C1) and (C2), which limit each agent to one task, we use the usage count vectors N_s_ and N_e_. To manage constraints (C3) and (C4), which limit the number of agents per target, we use the target assignment count vectors $N_{TS}$ and $N_{TE}$. These variables are formally defined in Equation (12).

(12) $$\left\{\begin{array}{c} N_{S\;}{=[n}_{S}{\left(i)\right]}_{1\times S}\\ N_{E}{=[n}_{E}{\left(j)\right]}_{1\times E}\\ N_{\mathrm{TS}}{=[\mathrm{nt}}_{S}{\left(k)\right]}_{1\times T}\\ N_{\mathrm{TE}}{=[\mathrm{nt}}_{E}{\left(k)\right]}_{1\times T} \end{array}\right.$$

During the iterative allocation process, these constraint variables guide the algorithm according to the following rules:

(1) Once a sensor i is assigned (i.e., when $n_{S}(i)\;=\;1$), it is removed from the set of available sensors for subsequent assignments.

(2) Similarly, once an effector agent j is assigned (i.e., when $n_{E}(j)\;=\;1$), it is removed from the set of available effectors.

(3) Once a target k reaches its maximum sensor capacity (i.e., when $nt_{S}(k)\;=\;m_{k}$), it is no longer considered for further sensor assignments.

(4) Once a target k reaches its maximum effector capacity (i.e., when $nt_{E}(k)\;=\;n_{k}$), it is no longer considered for further effector assignments.

### 5.3. Marginal Return Calculation

To solve the S-ETA mathematical programming model, this paper adopts the concept of “marginal return,” a principle borrowed from economics. In this context, the marginal return of a sensor–effector–target triad is the additional increase to the objective function (Total Expected Value) gained by adding that single assignment to the current plan.

The marginal return for every potential triad is calculated and stored in a matrix $\Delta ={\left[\delta_{ijk}\right]}_{S\times E\times T}$. The element $\delta_{ijk}$ represents the marginal return of adding the specific triad (i,j,k) to the current allocation scheme. It is computed as shown in Equation (13):

(13) $$\left\{\begin{array}{c} u_{1}(k)=v_{k}\left[1-P_{\mathrm{mis}}(k)\right]\left[1-Q_{\mathrm{mis}}(k)\right]\\ u_{2}(i,j,k)=v_{k}[1-P_{mis}(k){\left(1-p_{ik}\right)}^{y_{ik}}]\times [1-Q_{mis}(k){\left(1-q_{jk}\right)}^{z_{jk}}]\\ \delta_{ijk}=u_{2}(i,j,k)-u_{1}(k) \end{array}\right.$$

where the terms are defined as follows:

$u_{1}(k)\;$ represents the current expected value of servicing target k before the new triad is added.

$u_{2}(i,j,k)\;$ represents the potential new expected value of servicing target k after the triad (i, j, k) is included.

$\delta_{ijk}$ is the marginal return, calculated as the difference between the potential and current expected values.

$P_{mis}(k)$ is the probability that the group of sensors currently assigned to target k fails to achieve successful tracking.

$Q_{mis}(k)$ is the probability that the group of effector agents currently assigned to target k fails to service it successfully.

The pseudo-code of the designed algorithm is presented in Algorithm 1.
**Algorithm 1** MRBHA**Input:** S, E, T, V, P, Q, m_k_, n_k_
 
**Output:** X, Y, Z, J(Y, Z) 
 
1:  **// Initialization** 2:  X, Y, Z ← Initialize with zeros 3:  N_s_, N_e_, N_TS_, N_TE_ ← Initialize with zeros 4:  **for** each target k ∈ T **do** 5:     P_mis_(k) ← 1 6:     Q_mis_(k) ← 1 7:  **end for** 8:  CandidateTriples ← Generate all possible (i, j, k) triads 9:  **// Main Iterative Loop** 10: **while** CandidateTriples is not empty **do** 11:    max *δ ← -∞*
*12:    best_triad_ ← null*
*13:    **// Calculate marginal return for all currently valid candidates***
*14:    **for** each triad (i, j, k) in CandidateTriples **do***
*15:       δ*_ijk_ ← CalculateMarginalReturn(i, j, k, P_mis_, Q_mis_, V, P, Q) 16:       **if** δ*_ijk_ > max*δ **then** 17:         max*δ ← δ*_ijk_ 18:         best_triad_ ← (i, j, k) 19:       **end if** 20:    **end for** 21:    **// If no beneficial assignment is found, terminate** 22:    **if** max δ ≤ 0 **then** 23:       **break** 24:    **end if** 25:    // Update state with the best found triad (i*, j*, k*) 26:    (i*, j*, k*) ← best_triad_*
*27:    X(i*, j*, k*) ← 1; Y(i*, k*) ← 1; Z(j*, k*) ← 1 28:    N_s_(i*) ← 1; N_e_(j*) ← 1; N_TS_(k*)++; N_TE_(k*)++ 29:    Update P_mis_(k*) and Q_mis_(k*) 30:    **// Prune the candidate list: remove all newly infeasible triads** 31:    Remove all triads from CandidateTriples containing sensor i* or effector j* 32:    **if** N_TS_(k*) ≥ m_k_* **then** 33:       Remove all triads from CandidateTriples containing target k* for sensor assignment 34:    **end if** 35:    **if** N_TE_(k*) ≥ n_k_* **then** 36:       Remove all triads from CandidateTriples containing target k* for effector assignment 37:    **end if** 38: **end while** 39: **// Final Calculation** 40: J(Y, Z) ← CalculateTotalExpectedValue(Y, Z, V, P, Q) 41: **return** X, Y, Z, J(Y, Z)

### 5.4. Theoretical Analysis

To provide a more rigorous foundation for the proposed MRBHA, this section discusses its key theoretical properties, including computational complexity, convergence, and optimality guarantees.

#### 5.4.1. Computational Complexity

The computational complexity of MRBHA is primarily driven by the main iterative loop (lines 10–38 in Algorithm 1). Let S, E, and T denote the number of sensors, effectors, and targets, respectively.

The initial number of candidate triads is S×E×T.

In each iteration, the algorithm must calculate the marginal return for all remaining valid triads. In the worst case, this is $O\left(S\times E\times T\right)$.

The loop executes at most $min\left(S,E\right)$ times, as at least one sensor and one effector are removed from the available pool in each successful assignment iteration.

Therefore, the worst-case computational complexity of MRBHA is approximately $O\left(min\left(S,E\right)\times S\times E\times T\right)$. This pseudo-polynomial complexity makes the algorithm computationally efficient and tractable for the moderately sized problems typical in real-time UAV operations, where exact methods would be infeasible.

#### 5.4.2. Convergence

As a greedy heuristic operating on a finite set of discrete choices, MRBHA is guaranteed to converge. The algorithm terminates under one of two conditions:

1. All resources (sensors or effectors) have been allocated.
2. No remaining candidate triad yields a positive marginal return (max δ ≤ 0).

Since each iteration removes at least one sensor and one effector from the candidate pool, the total number of iterations is finite and bounded by $min\left(S,E\right)$. The objective function J(Y,Z) is monotonically non-decreasing, ensuring stable progression toward a final solution.

#### 5.4.3. Optimality Guarantees

MRBHA is a heuristic algorithm designed to find high-quality solutions in polynomial time, and as such, it does not provide a formal guarantee of finding the global optimum. The S-ETA problem is a complex combinatorial optimization problem, and finding a provably optimal solution would require exhaustive search methods that are computationally intractable for non-trivial problem sizes.

However, the algorithm’s design, based on the principle of maximizing marginal return, is a powerful greedy strategy. By always selecting the assignment that provides the greatest immediate increase to the Total Expected Value, MRBHA is structured to rapidly navigate the solution space toward regions of high quality. While it may converge to a local optimum, its holistic evaluation of the sensor–effector–target triad at each step is specifically designed to capture synergistic effects that simpler greedy methods would miss, thereby producing solutions that are empirically shown to be near-optimal and substantially better than standard baselines.

## 6. Computational Experiments and Analysis

### 6.1. Experimental Scenario Setup

(1) Generation of Target Value Vector: For the simulation scenario, a target value vector was generated to represent the priority of each target. To simulate a dynamic operational environment where target importance can vary, a random function was used to assign these values. Table 1 lists the generated target values used in the experiment.

(2) Generation of sensor capability matrix *P*,

(14) $$p_{ik}=p_{L}+(p_{H}-p_{L})\cdot rand$$

where $p_{L}$ and $p_{H}$ are predefined constants, $0\;<\;p_{L\;}<\;p_{H}\;<\;1$, reflecting the lower and upper limits of the sensor performance, respectively, and rand is the random number generation coefficient, rand∈[0,1]; taking $p_{L}=0.85$, $p_{H}=0.96$, the resulting sensor capability matrix is shown in Table 2.

For example, Table 2 indicates that the probability of sensor S1 successfully detecting and tracking target T1 is 0.93, while its probability for target T2 is 0.86. Similarly, the probability for sensor S2 to successfully detect and track target T6 is 0.85.

It is important to note that the sensor capability value, *p_i__k_*, serves as a comprehensive metric for the functional relevance of sensor i to target k. This probability is not merely a measure of detection; it is intended to be a composite score that can encapsulate a variety of factors, including the sensor’s modality (e.g., EO/IR, SAR), its resolution, its tolerance to environmental conditions, and its specific suitability for the target type. In a real-world application, these values would be derived from sensor specifications and empirical performance data, effectively providing the “weightage” that reflects each sensor’s importance to mission success.

(3) Generation of the Effector Effectiveness Matrix *Q*.

(15) $$q_{ik}=q_{L}+(q_{H}-q_{L})\cdot rand$$

where $q_{L}$ and $q_{H}$ are predefined constants, $0\;<q_{L\;}<\;q_{H}\;<\;1$, reflecting the lower and upper limits of Effector Agent performance, respectively, are taken as $q_{L}=0.80$, $q_{H}=0.98$. The generated effector agent effectiveness matrix is shown in Table 3.

Table 3 indicates that, for example, the probability that effector agent E1 is effective against target T1 is 0.81, its probability against target T2 is 0.94, and the probability that effector agent E2 is effective against target T6 is 0.82.

(4) Generation of $m_{k}$ and $n_{k}$: The number of sensors and effector agents assigned to a single target is set to one, two, or three depending on the value or priority (v_k_) of that target, as shown in Equations (16) and (17), respectively.

(16) $$m_{k}=\left\{\begin{array}{ll} 1, & \mathrm{if}\;0<v_{k}\leq 80\\ 2, & \mathrm{if}\;80<v_{k}\leq 90\\ 3, & \mathrm{if}\;90<v_{k}\leq 100 \end{array}\right.$$

(17) $$n_{k}=\left\{\begin{array}{ll} 1, & \mathrm{if}\;0<v_{k}\leq 50\\ 2, & \mathrm{if}\;50<v_{k}\leq 90\\ 3, & \mathrm{if}\;90<v_{k}\leq 100 \end{array}\right.$$

The maximum number of sensors and effector agents corresponding to different targets according to Equation (16) and Equation (17) is shown in Table 4.

Table 4 provides an example of these constraints. For instance, for target T1, the maximum number of assignable sensors is two, and the maximum number of assignable effector agents is two. For target T2, these limits are three for both sensors and effectors.

### 6.2. Assignment Results and Analysis

The corresponding data can be derived as follows, Table 5 shows the sensor–target matching matrix **Y**, and Table 6 shows the effector agent–target matching matrix **Z**.

From Table 5 and Table 6, the sensor–effector agent–target matching scheme is shown in Table 7.

**Figure 10:**
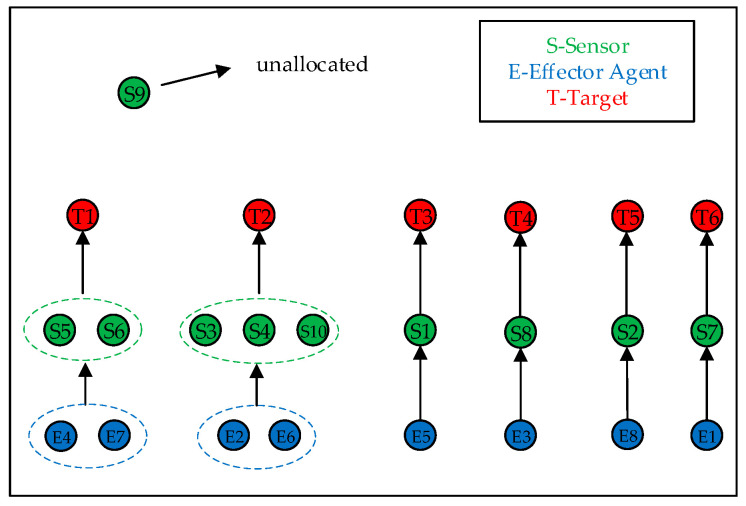
Visualization of the final assignment scheme.

In order to visualize the sensor–effector agent–target matching scheme more, the relevant allocation diagram is made, as shown in Figure 10.

Therefore, the optimal Mission Plan for incoming targets is shown in Table 8.

The final assignment plan, derived from the algorithm, is visualized in Figure 10. The solution successfully allocates resources to all six targets. For example, to service target T1, the plan assigns sensor agents S5 and S6, along with effector agents E4 and E7. This collaborative assignment strategy is applied across all targets to maximize the overall mission objective. Notably, sensor agent S9 remains unallocated, indicating efficient resource utilization based on the calculated marginal returns.

### 6.3. Comparative Analysis

To rigorously evaluate the performance of the proposed Marginal Return-Based Heuristic Algorithm (MRBHA), we conducted a comparative analysis against two baseline algorithms under the same experimental conditions described in Section 6.1.

Baseline 1: Random Assignment (RA): This algorithm randomly assigns available sensor–effector–target triads until resource constraints are met. It serves as a lower-bound benchmark to demonstrate the value of an intelligent allocation strategy.

Baseline 2: Simple Greedy (SG): This algorithm decouples the assignment process. It first greedily assigns the best available sensor to each target based on the highest tracking probability ($p_{ik}$). Then, it greedily assigns the best available effector to each target based on the highest servicing probability ($q_{jk}$). This method does not consider the synergistic, marginal return of the complete triad.

The performance of each algorithm was evaluated based on two key metrics:

Total Expected Value ($J\left(Y,\;Z\right)$): The primary objective function value, indicating the overall quality and effectiveness of the assignment plan. A higher value is better.

Computation Time (s): The time required for the algorithm to produce a solution, measuring its computational efficiency. A lower value is better, especially for dynamic scenarios.

**Figure 11:**
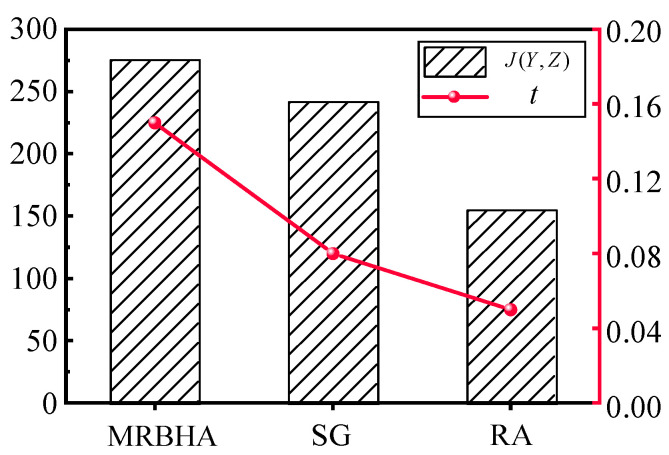
Comparison of Total Expected Value achieved by different algorithms.

The Performance comparison of different algorithms in terms of Total Expected Value (left axis) and Computation Time (right axis). The specific results are shown in Table 9 and Figure 11.

The proposed MRBHA significantly outperforms both baseline methods. The experimental results clearly demonstrate the superiority of the proposed MRBHA.

Effectiveness: MRBHA achieved a Total Expected Value of 275.4, which is approximately 14% higher than the Simple Greedy algorithm and 77% higher than Random Assignment. This significant improvement validates the core hypothesis of this paper: that optimizing based on the marginal return of the complete sensor–effector–target triad yields substantially better solutions than decoupled or random approaches. The SG algorithm, while better than random, fails to capture the synergistic effects, leading to sub-optimal global solutions.

Efficiency: A key finding is that MRBHA achieves its superior solution quality with remarkable computational efficiency. Despite its more sophisticated, holistic decision-making process compared to the baseline methods, it produced the optimal assignment in just 0.15 s. This demonstrates that the algorithm’s pseudo-polynomial complexity is highly practical for real-world scenarios. The minor increase in Computation Time over the much simpler greedy and random methods is an exceptionally small price to pay for the substantial (14−77%) gain in mission effectiveness. This result confirms that MRBHA strikes an excellent balance, providing near-optimal solutions without sacrificing the computational feasibility required for dynamic, time-sensitive environments.

In summary, the comparative analysis confirms that our proposed MRBHA provides a highly effective and efficient solution to the S-ETA problem, striking an excellent balance between solution optimality and computational speed.

The underlying reason for MRBHA’s superior performance lies in its core design philosophy. Unlike the SG algorithm, which makes locally optimal choices for sensors and effectors in isolation, MRBHA evaluates the holistic, synergistic contribution of the entire sensor–effector–target triad. By calculating the marginal return, it quantifies the precise value added by each potential assignment to the global objective function. This allows it to identify and prioritize assignments that may seem sub-optimal in one stage (e.g., a slightly weaker sensor) but unlock a disproportionately high value in a subsequent stage (e.g., enabling a highly effective effector), an emergent synergy that simpler greedy methods are blind to.

### 6.4. Scalability Analysis

To evaluate the scalability and robustness of the proposed MRBHA, an additional experiment was conducted on a large-scale mission scenario. This scenario was designed to test the algorithm’s performance under a significantly higher computational load, reflecting more complex operational environments.

**Figure 12:**
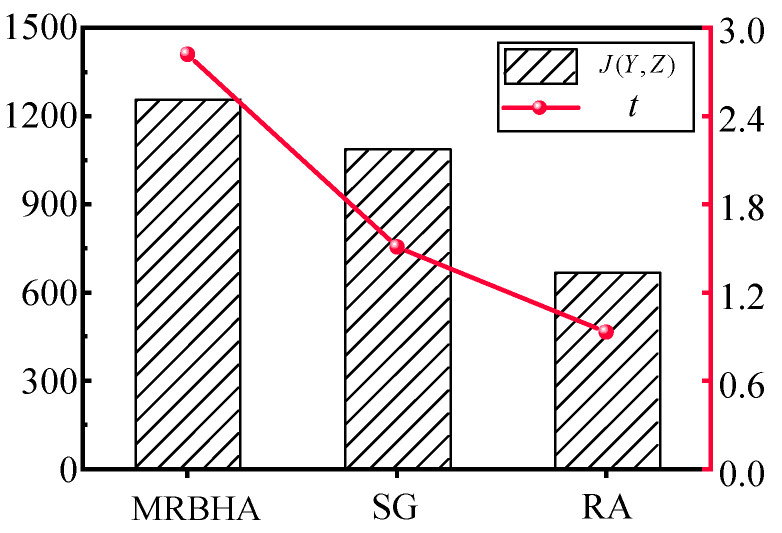
Comparison of Computation Time in the large-scale scenario.

The large-scale scenario was configured with 50 targets (T = 50), 30 sensor agents (S = 30), and 20 effector agents (E = 20). All capability matrices and value vectors were randomly generated following the same distributions described in Section 6.1. The MRBHA, Simple Greedy (SG), and Random Assignment (RA) algorithms were all executed on this larger problem set. The performance results are summarized in Table 10 and Figure 12.

The results from the large-scale experiment further validate the superiority and robustness of the MRBHA.

1. Sustained Performance Advantage: In the more complex scenario, MRBHA not only maintained its performance lead but arguably demonstrated its synergistic advantage more clearly. It outperformed the SG algorithm by approximately 15.4% and the RA algorithm by 87.3%, showing that its ability to capture holistic triad value becomes even more critical as the number of combinatorial possibilities increases.

2. Practical Computational Time: While the Computation Time for MRBHA increased to 2.82 s, this remains well within a practical range for near-real-time, event-triggered re-planning. For strategic and operational-level decision-making, a response time of a few seconds to generate a globally aware, high-quality solution is highly acceptable. This confirms that the algorithm scales effectively, providing a robust solution without becoming computationally prohibitive.

### 6.5. Discussion on Practical Implications

While our simulations with randomly generated data effectively validate the algorithmic advantages of the proposed framework, transitioning to a real-world deployment as depicted in Figure 7 presents several practical challenges. A key requirement would be that the population of the capability matrices (P and Q) and target values (V) use high-fidelity, real-world data derived from field tests, sensor specifications, and real-time intelligence, rather than statistical distributions.

Furthermore, our current model simplifies certain operational complexities, most notably the explicit modeling of temporal dynamics. The S-ETA formulation presented here is a static, or “snapshot-based,” optimization that assumes assignments can be made near-instantaneously without considering factors such as UAV travel time to targets, the duration of sensing or effecting tasks, or specific mission deadlines. This was a deliberate modeling choice to first isolate and solve the core challenge of synergistic, multi-stage resource allocation. However, for real-world deployment, integrating these spatio-temporal constraints is critical. Other factors not explicitly modeled, such as communication latency, battery constraints, and environmental dynamics (e.g., wind, obstacles), would also need to be integrated for robust performance.

Another aspect of dynamism relates to how the system adapts to real-time changes in the operational environment—or, as the reviewer aptly puts it, changes in the “graph.” This includes events like the sudden appearance of new high-priority targets, the loss of a UAV, or the degradation of a communication link. Our proposed approach addresses this form of dynamism through event-triggered re-planning. The high computational efficiency of the MRBHA (producing a solution in 0.15 s in our tests) is a critical feature in this context. It is not designed to run only once, but to be executed rapidly whenever a significant event occurs, generating a new, globally aware assignment plan for the updated set of agents and tasks.

A note on advanced baseline comparisons is also warranted. While this study used greedy and random algorithms as foundational benchmarks, we acknowledge the existence of more advanced approaches like multi-agent reinforcement learning (MARL) and distributed optimization. The choice to develop a centralized heuristic was deliberate for this problem context. MARL approaches, while powerful, often require extensive offline training, face challenges with sample efficiency, and may struggle to generalize scenarios with dynamically changing numbers of agents and targets. Distributed algorithms are highly effective in decentralized systems with limited communication but may converge to sub-optimal solutions from a global perspective. Our MRBHA, by contrast, is a training-free, centralized method designed to find high-quality, globally aware solutions with very low computational overhead, making it particularly suitable for scenarios where a central decision-making node is available and rapid re-planning is paramount.

## 7. Conclusions

In this paper, we introduced and validated a novel hierarchical framework and a Marginal Return-Based Heuristic Algorithm (MRBHA) to address the complex Sensor–Effector–Target Assignment problem in heterogeneous UAV networks. Our principal finding is that by explicitly modeling the multi-stage mission workflow and capturing the synergistic effects between agents, our system achieves significant performance gains over traditional, decoupled approaches. The experimental results quantitatively confirmed this, showing that the MRBHA yielded a total mission value approximately 14% higher than a simple greedy strategy and 77% higher than random assignment.

This work demonstrates that a structured, hierarchical approach is not merely a conceptual exercise but a practical tool for unlocking superior performance in multi-agent systems. The computational efficiency of the MRBHA further suggests its suitability for dynamic, time-sensitive operational environments.

A primary direction for future research will be to extend the framework to explicitly incorporate spatio-temporal dynamics. This involves integrating constraints such as UAV travel time, task execution durations, and target deadlines directly into the optimization model, transforming the problem into a dynamic vehicle routing and scheduling challenge. Furthermore, developing event-triggered re-planning mechanisms to enhance the framework’s adaptability in uncertain environments remains a promising direction. Validating the proposed system through hardware-in-the-loop simulations will also be a crucial step towards real-world application.

Furthermore, a comprehensive comparative study against advanced state-of-the-art benchmarks, including multi-agent reinforcement learning (MARL) and distributed optimization algorithms (e.g., Consensus-Based Bundle Algorithm), is a key priority for future work. This will allow for a more nuanced understanding of the trade-offs between our heuristic’s computational efficiency and the potential performance gains of more complex, learning-based or decentralized methods.

## Figures and Tables

**Table 1:** Generated target values.

	T1	T2	T3	T4	T5	T6
V	83	96	42	14	50	35

**Table 2:** Sensor capability matrix.

	T1	T2	T3	T4	T5	T6
S1	0.93	0.86	0.96	0.9	0.87	0.9
S2	0.89	0.93	0.91	0.9	0.96	0.85
S3	0.87	0.9	0.93	0.9	0.91	0.9
S4	0.94	0.95	0.85	0.88	0.86	0.96
S5	0.89	0.87	0.93	0.88	0.92	0.88
S6	0.95	0.87	0.9	0.93	0.95	0.88
S7	0.93	0.85	0.95	0.95	0.89	0.95
S8	0.91	0.92	0.93	0.93	0.9	0.88
S9	0.87	0.9	0.96	0.9	0.95	0.9
S10	0.89	0.94	0.92	0.86	0.86	0.89

**Table 3:** Effector agent effectiveness matrix.

	T1	T2	T3	T4	T5	T6
E1	0.81	0.94	0.89	0.83	0.86	0.94
E2	0.83	0.91	0.81	0.87	0.86	0.82
E3	0.82	0.89	0.86	0.91	0.83	0.87
E4	0.95	0.89	0.85	0.84	0.96	0.82
E5	0.8	0.93	0.9	0.84	0.8	0.96
E6	0.97	0.98	0.82	0.9	0.88	0.9
E7	0.94	0.87	0.86	0.9	0.87	0.9
E8	0.85	0.89	0.96	0.84	0.93	0.86

**Table 4:** Maximum number of assignable agents per target.

	T1	T2	T3	T4	T5	T6
S	2	3	1	1	1	1
E	2	3	1	1	1	1

**Table 5:** Sensor–target matching matrix **Y**.

	T1	T2	T3	T4	T5	T6
S1	0	0	1	0	0	0
S2	0	0	0	0	1	0
S3	0	1	0	0	0	0
S4	0	1	0	0	0	0
S5	1	0	0	0	0	0
S6	1	0	0	0	0	0
S7	0	0	0	0	0	1
S8	0	0	0	1	0	0
S9	0	0	0	0	0	0
S10	0	1	0	0	0	0

**Table 6:** Effector agent–target matching matrix **Z**.

	T1	T2	T3	T4	T5	T6
E1	0	0	0	0	0	1
E2	0	1	0	0	0	0
E3	0	0	0	1	0	0
E4	1	0	0	0	0	0
E5	0	0	1	0	0	0
E6	0	1	0	0	0	0
E7	1	0	0	0	0	0
E8	0	0	0	0	1	0

**Table 7:** Final assignment scheme.

	T1	T2	T3	T4	T5	T6
S	S5, S6	S3, S4, S10	S1	S8	S2	S7
E	E4, E7	E2, E6	E5	E3	E8	E1

**Table 8:** Summary of the optimal assignment plan.

Target	Assigned Sensor	Assigned Effector
T1	5, 6	4, 7
T2	3, 4, 10	2, 6
T3	1	5
T4	8	3
T5	2	8
T6	7	1

**Table 9:** Performance comparison of different algorithms.

Algorithm	Total Expected Value (J(Y,Z))	Computation Time (s)
MRBHA (Proposed)	275.4	0.15
Simple Greedy (SG)	241.8	0.08
Random Assignment (RA)	155.2	0.05

**Table 10:** Performance comparison in a large-scale scenario.

Algorithm	Total Expected Value (J(Y,Z))	Computation Time (s)
MRBHA (Proposed)	1255.4	2.82
Simple Greedy (SG)	1088.2	1.51
Random Assignment (RA)	670.1	0.93

## Data Availability

The data presented in this study are available within the article. The simulation experiments were conducted based on the parameters and models described herein.

## Appendix A

**Table A1:** Description of symbols.

$Y={\left[y_{ik}\right]}_{S\times T}$	Sensor-target assignment matrix, if the ith sensor is assigned to the kth target, then $y_{ik}=1$, otherwise $y_{ik}=0$ i=1,2,⋯,S, k=1,2,⋯,T
$Z={\left[z_{jk}\right]}_{E\times T}$	Effector Agent -target assignment matrix, if Effector Agent j is assigned to target k then $z_{jk}=1$, otherwise $z_{jk}=0$, j=1,2,⋯,E
$P_{serv}(k)$	successfully servicing the kth target
$P_{s}(k)$	Probability of success of a sensor that detects, localizes and tracks the kth target
$P_{e}(k)$	Probability that the selected Effector Agent is effective against the kth target.
$P={\left[p_{ik}\right]}_{S\times T}$	Sensor capability matrix, where $p_{ik}$ denotes the probability of sensor ith successfully detecting target kth
$Q={\left[q_{jk}\right]}_{E\times T}$	Effector effectiveness matrix, $q_{jk}$, represents the probability that the jth Effector Agent will be effective against the kth target.
J(Y,Z)	Total expected value of the targets serviced by the assignment plan
$V={\left[v_{k}\right]}_{1\times T}$	Target value vector, where $v_{k}$ represents the priority or value of the kth target.
$m_{k}$	Maximum number of sensors that can be assigned to the kth target at one time
$n_{k}$	Maximum number of Effector s that can be assigned to the kth target at any one time
$X={\left[x_{ijk}\right]}_{S\times E\times T}$	Sensor- Effector -Target Assignment Matrix, if the ith sensor and jth Effector are assigned to the kth target, then $x_{ijk}=1$, otherwise $x_{ijk}=0$
$N_{s}={\left[n_{S}(i)\right]}_{1\times S}$	Vector of sensor usage counts to record how often each sensor was called in the actual configuration
$N_{E}{=[n}_{E}{\left(j)\right]}_{1\times E}$	Vector of Effector Agent usage counts to record how often each Effector Agent is called in the actual configuration
$N_{\mathrm{TS}}{=[\mathrm{nt}}_{S}{\left(k)\right]}_{1\times T}$	Used to record the number of sensors assigned to each target in the actual distribution plan
$N_{TE}{=[\mathrm{nt}}_{E}{\left(k)\right]}_{1\times E}$	Used to record the number of Effector Agent s allocated to each target in the actual distribution plan
$\Delta ={\left[\delta_{ijk}\right]}_{S\times E\times T}$	Marginal return value of the kth objective after adding the additional allocation triad (i,j,k) to the allocation scheme
$U_{1}={\left[u_{1}(k)\right]}_{1\times T}$	Vector of expected value of t servicing for each target after the previous allocation, $u_{1}(k)\;$denotes the expected value of the kth target serviced after the previous allocation
$U_{2}={\left[u_{2}(i,j,k)\right]}_{S\times E\times T}$	Matrix of expected value of servicing for each sensor-Effector Agent -target triad after allocation, $u_{2}(i,j,k)\;$represents the expected value of the kth target serviced after adding triad (i,j,k) to the allocation scheme
$P_{mis}(k)$	Probability that all sensors assigned to the kth target fail to track it stably
$Q_{mis}(k)$	Probability that the group of effector agents assigned to the kth target fails to service it successfully.
